# Clinical outcomes of patients with advanced EGFR mutated nonsquamous cell lung carcinoma treated at a tertiary care hospital

**DOI:** 10.3332/ecancer.2025.1960

**Published:** 2025-08-07

**Authors:** Faiza Ahmed, Aqsa Amjad, Eman Anwar, Mohammad Saad Saleem Naviwala, Warda Saleem, Nawazish Zehra, Munira Moosajee, Yasmin Abdul Rashid

**Affiliations:** 1 Department of Oncology, Section of Medical Oncology, Aga Khan University Hospital, Sindh, Karachi 748000, Pakistan; 2 Aga Khan Medical College, Aga Khan University Hospital, Sindh, Karachi 748000, Pakistan

**Keywords:** epidermal growth factor receptor (EGFR), advanced lung cancer, progression-free survival, overall survival

## Abstract

**Background:**

Epidermal growth factor receptor (EGFR)-mutated advanced adenocarcinoma of the lung is among the most prevalent mutation types. The treatment paradigm for this condition is rapidly evolving. This study focuses on the clinical outcomes in resource-limited settings. The findings aim to guide treatment strategies for such populations.

**Methods:**

A retrospective analysis was conducted on 51 patients aged over 18 years with EGFR-positive nonsquamous lung carcinoma treated at Aga Khan University Hospital between January 2017 and December 2021. Data were collected using nonprobability consecutive sampling and reviewed retrospectively from patient records. Statistical analyses were performed using Statistical Package for Social Science version 20.0. Continuous data were analysed using an independent sample T-test, while categorical data were assessed using the Cox regression test. Kaplan–Meier survival curves were generated to evaluate overall survival (OS) and progression-free survival (PFS), and the log-rank test was used to compare median PFS. A *p*-value of <0.05 was considered statistically significant for all analyses.

**Results:**

The median age of the cohort was 60 years, with a gender distribution of 56% females and 44% males. A total of 98% of the patients presented with de novo stage IV lung adenocarcinoma. All patients had EGFR-mutated adenocarcinoma, and the majority (80.4%) were nonsmokers. Mutational analysis revealed the following: Exon 19 deletion in 56.9% of patients, Exon 20 insertion in 9.8%, Exon 21 mutations in 19.6%, compound mutations in 7.8%, other mutations in 2.0% and the de novo T790M mutation in 3.9%. Among the cohort, 82% received at least one line of EGFR tyrosine kinase inhibitors (TKIs). The median PFS with TKIs was 15 months, and the median OS with first-line TKIs was 38 months.

**Conclusion:**

This study demonstrates that EGFR-targeted therapy, when used in a first-line setting, significantly improves OS and PFS in this population. Further research is warranted to optimise treatment strategies, particularly in resource-limited settings.

## Introduction

Lung cancer is the most common cancer worldwide, in 2018, lung cancer diagnoses totaled 2 million worldwide, comprising 11.6% of all cancer cases [1]. According to GLOBOCAN in 2020, there were 10,538 new cases of lung cancer across all genders and age groups, accounting for around 5.9% of all new cancer cases in Pakistan [2]. Globally, outcomes for lung cancer remain poor. Due to its often asymptomatic early stages, many patients are diagnosed at an advanced stage, which makes curative treatment unlikely. Statistics indicate that more than half of individuals diagnosed with lung cancer succumb within 1 year of their diagnosis, with a 5-year survival rate of approximately 17.8% [3]. It also stands as the foremost cause of cancer-related deaths among men. Additionally, in women, lung cancer exhibits high mortality rates, ranking second only to breast cancer [4].

Nonsmall cell lung cancer (NSCLC) is one of the most common histological subtypes of lung cancer, making about 85% of all cases [5]. NSCLC is then further divided into three types: squamous-cell carcinoma, adenocarcinoma and large-cell carcinoma [5]. Squamous-cell carcinoma, representing 25%–30% of lung cancer cases, originates from early squamous cells in the bronchial tubes and is strongly linked to smoking. Adenocarcinoma, the most common type (40% of cases), arises from airway epithelial cells, often in the lung periphery, and tends to grow slowly. Large cell carcinoma (5%–10% of cases) lacks squamous or glandular features and typically begins centrally, possibly spreading to nearby lymph nodes and distant organs [6]. Our study will centre on nonsquamous lung carcinoma, encompassing on adenocarcinoma as the subject of our investigation.

NSCLC is not a single disease. The identification of various oncogenic mutations has substantially transformed the treatment and prognosis of lung cancer. It has been observed that targeted therapies for these mutations are less toxic and more effective than the previously used platinum-based chemotherapy [7]. The primary and earliest actionable mutation identified is the epidermal growth factor receptor (EGFR). EGFR, part of the human epidermal receptor family, plays a pivotal role in cell signaling. Upon binding with ligands like epidermal growth factor and transforming growth factor-alpha, EGFR undergoes conformational changes. These changes influence various pathways including the RAS/RAF/MAPK, PI3K/AKT and JAK/STAT pathways. Activation of these pathways promotes cell proliferation through mitosis and suppresses apoptosis, essential processes for normal cell growth [8]. EGFR is one of the most common mutations identified in NSCLC, incidence in Asian population about 50% while in the Western population, it is 10% [9].

Several activating mutations are recognised in EGFR in NSCLC, including Class I exon 19 in-frame deletions (accounting for 44% of all EGFR mutations), Class II single amino acid changes (such as L858R accounting for 41%, G719 for 4% and other missense mutations for 6%) and Class III exon 20 in-frame duplications or insertions (constituting 5%) [10]. EGFR tyrosine kinase inhibitors (TKI) have shown better outcomes. However, acquired resistance does develop from first-generation (Erlotinib and gefitinib) and second-generation (Afatinib and dacomitinib) TKI. EGFR T790 gatekeeper mutation develops after 9 to 15 months [11]. Osimertinib is the third-generation TKI which have the potential to cross blood–brain barrier and seem to be effective against T790 mutation. Osimertinib is a widely approved treatment option in first-line settings in advanced EGFR mutated nonsmall cell lung carcinoma. Flaura trial is one of the landmark study where osimertinib when compared with earlier generation TKIs shows benefit in progression-free survival (PFS) of 8 months with better central nervous system efficacy [12]. Furthermore, the role of osimertinib combined with chemotherapy has been studied, showing an additional PFS benefit of 8.8 months. This benefit was particularly observed in individuals with central nervous system disease [13].

Multiple studies have reported the benefits of adding antiangiogenic agents, such as bevacizumab and ramucirumab, to first-generation TKIs like erlotinib. The data suggest that the addition of antiangiogenic agents to TKIs improves PFS. The benefit with bevacizumab was 6 months, while with ramucirumab, it was 7 months [14]. A current pressing issue is the resistance to osimertinib. Studies are being conducted to identify the mechanisms of resistance and to develop treatments to overcome it. EGFR C797S is the most common acquired mutation after progression on osimertinib. New fourth-generation TKI is being studied to overcome it [15]. The current treatment landscape for EGFR-mutated advanced NSCLC has evolved. With the advent of new treatment combinations, it is now possible to select the most appropriate option on an individualised basis.

In our tertiary care centre, the protocol for managing advanced nonsquamous lung cancer patients harboring EGFR-positive mutations involved employing a range of TKIs. Following disease progression, a sequential treatment approach is adopted, which incorporates chemotherapy, immunotherapy and anti-angiogenic agents. Given the emerging evidence indicating ethnic disparities in treatment responses, there is an increasing need to investigate the diverse clinical presentations observed within the Pakistani population. Hence, the primary aim of our study is to comprehensively evaluate the clinical manifestations, therapeutic outcomes and molecular profiles of advanced EGFR-positive nonsquamous lung cancer patients within our tertiary care setting in Pakistan. Our specific objectives include assessing the correlation between distinct clinical phenotypes and the types of EGFR mutations, tumour localisation patterns and responses to various TKIs as well as subsequent lines of therapy. Additionally, we also aim to compare data from Western cohorts, seeking to identify any divergent trends. Through this comparison, our study endeavors to optimise treatment strategies based on localised clinical experiences. Consequently, this study would contribute to refining therapeutic protocols tailored to the unique characteristics of the South Asian population. Ultimately, such advancements would facilitate the progress of personalised medicine in the management of advanced nonsquamous lung cancer.

## Methods

### Participants and study settings

All patients (age >18 years) with EGFR-positive nonsquamous lung carcinoma treated at the Department of Oncology, Aga Khan University Hospital (AKUH) from January 2017 to December 2021 were included in the study through nonprobability consecutive sampling. A retrospective review of patient records was performed, and data were entered into pre-designed Performa’s. Patients treated for other diagnosed malignancies apart from lung carcinoma or those treated outside AKUH during the study period were excluded.

### Data collection

Baseline data were collected on gender, disease characteristics and Eastern Cooperative Oncology Group (ECOG) performance status. Risk factors such as smoking and family history of cancer were recorded. EGFR was checked by polymerase chain reaction in 47 patients while 4 were checked by next-generation sequencing. The type of targeted therapy involved oral EGFR-TKIs (Erlotinib 150 mg once a day, Afatinib 40 mg once a day and Osimertinib 80 mg once a day), and treatment response was assessed through radiological scans done periodically. PFS was defined as the time from the end of treatment to radiological or clinical disease progression. Overall survival (OS) was defined as the length of time from the date of diagnosis and till the date of death.

### Statistical analysis

The data were entered and analysed using the Statistical Package for Social Science version 20 (Chicago, Illinois). Categorical variables were analysed for frequency, percentage and graphical representations. For continuous data, we used an independent sample *T* test. Categorical data were analysed using Cox regression. Kaplan–Meier survival curves were generated to present patients’ OS, and the log-rank test was utilised to compare median PFS times. A *p*-value of < 0.05 was considered significant for all analyses.

### Ethical considerations

The study was conducted in compliance with the principles of the Declaration of Helsinki and the Good Clinical Practice guidelines. Ethical approval was obtained from the Ethics Review Committee at the Aga Khan University, Karachi, Pakistan (ID Number: 8039).

## Results

### Demographics

51 patient charts were reviewed, with a mean age of 60 years. The cohort consisted of 56% females and 43% males. At presentation, 41.2% of patients had disease involvement in the lung, pleura, brain and liver, and nearly 98% were diagnosed at stage IV. Regarding smoking history, 80.4% were nonsmokers, while 19.6% were smokers, with varying smoking intensities: 3.9% had 20 or more pack-years, 3.9% had 30 or more pack-years, 3.9% had 10 or more pack-years, 2.0% had 100 or more pack-years and 2.0% had an unknown number of pack-years (*p* = 0.00). A family history of cancer was reported by 11.8% of patients (*p* = 0.01), including reports of cancer in the mother, daughter, brother or general family history, with specific cancer types each accounting for 2.0%. The remaining 88.2% had no family history of cancer.

Performance status, measured using the ECOG scale, showed that 51.0% of patients had a status of 0–1, 27.5% had a status of 2, 17.6% had a status of 3 and 3.9% had a status of 4. Pleural fluid cytology was negative in 21.6% of patients, positive in 25.5% and not performed in 52.9%. Histological confirmation revealed that all patients had adenocarcinoma. Genetic analysis showed that 56.9% of patients had an Exon 19 deletion, 9.8% had an Exon 20 insertion, 19.6% had Exon 21 mutations, 7.8% had compound mutations, 2.0% had other mutations and 3.9% had the T790M mutation.

For targeted therapy, 35.3% of patients received Erlotinib, 2.0% received Afatinib, 45.1% received Osimertinib and 17.6% did not receive any targeted therapy. Dose reductions due to side effects were necessary for 11.8% of patients, while 72.5% did not require any reductions and 15.7% required no adjustments. Among those needing reductions, 74.5% did not have further adjustments, 2.0% had dose reductions of 10% to 29%, 9.8% had reductions of 30% to 50% and 13.7% had no additional reductions.

Regarding treatment response, 72.5% of patients experienced disease progression, 23.5% had stable disease and 3.9% did not have their response assessed (Table 1).

### Univariate analysis

In the univariate analysis, various variables were evaluated for their potential impact on the outcome, with a significance threshold set at 0.25. Performance Status (ECOG) exhibited a significance level of 0.471, indicating no significant association with the outcome. The odds ratio (Exp (B)) for this variable was 0.015, with a 95% confidence interval ranging from 0.000 to 1,327.440, reflecting a wide interval due to the low significance. Similarly, the type of mutation detected had a significance level of 0.725, suggesting it was not significantly associated with the outcome. The odds ratio was 0.178, with a 95% confidence interval extending from 0.000 to 2,712.892, indicating variability in the impact of mutation types. The type of targeted therapy also showed no significant association, with a significance level of 0.616 and an odds ratio of 434.450. The confidence interval for this variable was extremely broad, ranging from 0.000 to 8,953,863,135,600.588, highlighting considerable uncertainty in the effect estimate. The stage at diagnosis similarly had a significance level of 0.616, with an odds ratio of 0.048 and a 95% confidence interval from 0.000 to 6,887.566, reflecting wide variability. The location of the disease was not significantly associated with the outcome, evidenced by a significance level of 0.725 and an odds ratio of 0.178, with a 95% confidence interval from 0.000 to 2,712.892. Gender showed a significance level of 0.616, indicating no significant association, with an odds ratio of 434.450 and a confidence interval ranging from 0.000 to 8,953,863,135,600.588, suggesting high uncertainty. Finally, Pleural Fluid Cytology (Applicable if Fluid is Present) had a significance level of 0.464, with an odds ratio of 6.458 and a confidence interval from 0.044 to 947.343. Although the odds ratio implies a potential impact, the broad confidence interval reflects considerable uncertainty. Overall, none of the variables demonstrated significant associations with the outcome at the 0.25 significance level, and the wide confidence intervals for several variables indicate substantial variability in the effect estimates, suggesting a need for further investigation (Table 2).

### Progression free survival

**Figure 1.**
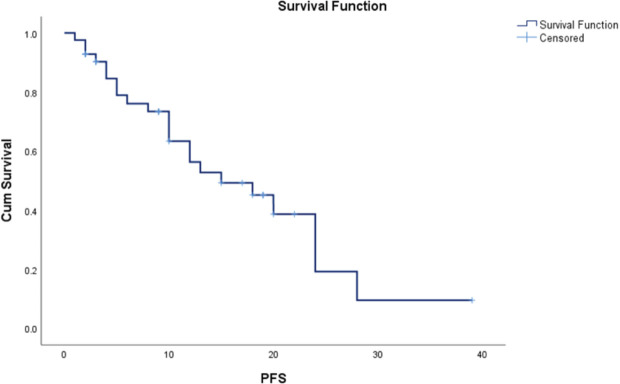
PFS.

The median PFS time is 15.0 months with a standard error of 4.062. The 95% confidence interval for this median estimate ranges from 7.038 to 22.962. This interval indicates the range within which the true median PFS time is expected to fall with 95% confidence. The median PFS time is a measure that divides the survival times into two equal halves, meaning that 50% of the study population experienced a PFS time shorter than 15.0 months and 50% experienced a survival time longer than this value (Figure 1).

### Overall survival

**Figure 2.**
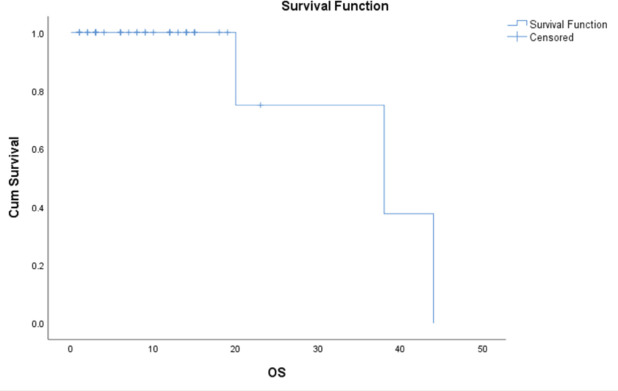
OS.

The median survival time is 38.000 months, with a standard error of 13.748. The 95% confidence interval for this median estimate ranges from 11.054 to 64.946. This interval indicates the range within which the true median survival time is expected to fall with 95% confidence. The median survival time is a measure that divides the survival times into two halves, meaning that 50% of the study population experienced a survival time shorter than 38.000 and 50% experienced a survival time longer than this value (Figure 2).

### Toxicity profile

Patients treated with EGFR-targeted kinase inhibitors demonstrated good overall tolerability. The only observed toxicity was the development of skin rashes. Of the cohort, six patients (11.7%) experienced skin rashes of varying severity. Grade III rashes were reported in five patients, necessitating dose reductions, while a single patient experienced a grade II rash, which did not require modification of the treatment regimen (Table 3).

## Discussion

Our study found that the median PFS and median OS for EGFR-positive nonsquamous lung cancer patients was 15 and 38 months, respectively, which is higher than what has been reported in the literature. For instance, a Peruvian cohort reported a median survival of 9.3 months [16], while a Portuguese cohort found it to be 12 months [17]. Similarly, a randomised controlled trial by the ECOG involving 1,207 patients reported a median OS of 8 months [18]. However, a Japanese cohort observed a median OS of 29.7 months [19], which is more consistent with our findings.

Our study was unable to find any significant factor that influenced the OS of these patients, factors such as gender, stage of disease, location of disease (the different metastatic locations), the type of EGFR mutation, pleural fluid cytology or the performance status as all these variables *p* values were greater than 0.05. In the past literature, however, there have been factors reported in the literature that seem to influence the OS in EGFR-positive NSCLC patients. A retrospective study conducted at 17 medical centres across Japan found on a multivariate final model Cox regression analysis, younger age (<75 years), no smoking history, histological diagnosis of adenocarcinoma, less advanced clinical stage, good performance status and major EGFR-activating mutation were identified as significant predictors of OS [19]. The factors that were found to be significantly affecting the OS in the Peruvian cohort were patient age (< 65 years), hemoglobin (> 12 g/dL), histological type (adenocarcinoma) and type of mutation (point mutation of codon 858) [20]. Another retrospective cohort found that types of metastases for example in the brain and bone were significantly associated with OS [20]; however, this factor was not significant in our study. On the other hand, another retrospective study found that sex, age, smoking habits and the EGFR exon mutation did not have a statistical impact on OS in the EGFR-mutated NSLC patients [21], this was like our study’s findings.

Almost every patient in our cohort received targeted therapy from the TKIs with 23 of our patients receiving Osimertinib while 18 received Erlotinib. Only one patient received conventional chemotherapy, so we were unable to make direct comparisons between chemotherapy and traditional TKIs. However, previous studies have shown that treatment with TKIs, such as erlotinib, improved OS to 10.4 months. Additionally, the median PFS was 4.8 months for patients who received TKI as a first-line treatment, compared to 2.9 months for those treated with chemotherapy. These studies have also reported a better quality of life in patients treated with erlotinib compared to those receiving chemotherapy. Moreover, the side effect profile of TKIs is generally more favourable than that of conventional chemotherapy [21, 22]. Furthermore, there was no impact on OS from different generations of TKI, 3rd Generation Osimertinib and 1st and 2nd generation TKI such as Erlotinib, this was a consistent finding with the literature. A Dutch nationwide real-world cohort found there was no observed difference in OS benefit for patients treated with the third-generation TKI Osimertinib as a first-line therapy compared to those receiving first- or second-generation TKIs [23]. Conversely, the FLAURA trial reported a PFS benefit of 8.7 months and an OS advantage of 6 months when first-line Osimertinib was compared to first-generation TKIs, such as gefitinib and erlotinib [12]. A recently published retrospective study conducted in Pakistan, involving a similar cohort of 37 patients, reported a 5.6-month improvement in outcomes with first-line TKIs compared to chemotherapy [24]. Even with a small number of patients, these results are quite similar to our study.

Our study has several limitations, including its retrospective design, which is prone to inherent biases. Data collection may have been affected by incomplete clinical records and missing information, potentially introducing selection bias. Moreover, since the study was conducted at a single healthcare facility, the results may not be generalisable to the wider population. The relatively small sample size also limited our ability to detect significant associations. Additionally, we could not compare outcomes with patients who received conventional chemotherapy, as only one of the participants in our cohort underwent such treatment.

Despite these limitations, to the best of our knowledge, this is the first Pakistani study to analyse OS associated with TKI therapy, offering valuable national evidence on survival factors in EGFR-positive NSCLC patients treated with TKIs as first-line therapy. Hence, our study could form the basis of upcoming clinical trials or longitudinal multicentre studies investigating prognostic factors affecting OS in EGFR-positive NSCLC patients.

## Conclusion

Our study identified a median PFS of 15 months and median OS of 38 months among EGFR-positive nonsquamous advanced nonsmall cell lung carcinoma patients, surpassing survival metrics reported in international cohorts. Although no specific factors significantly impacted survival in our analysis, this aligns with findings from other studies reporting no association between survival outcomes and variables. Furthermore, this review highlights the necessity of prospective, multicentre study to determine the pattern of EGFR-mutated lung cancer and how it adheres to existing treatments in order to develop a suitable strategy for the South Asian population.

## Conflicts of interest

All authors declare no competing financial interests.

## Funding

No funding has been used.

## Consent to participate

Due to the retrospective nature of the study, the need for informed consent was waived by the ERC of Aga Khan University Hospital, Karachi, Pakistan.

## Consent to publication

Not applicable.

## Ethics approval and consent to participate

This study was approved by Institutional Ethics Review Committee (ERC number: 2022-7963-22909) and all participants provided written informed consent.

## Author contributions

FA, YAR, AA, EA: Designed and wrote the manuscript

AA, EA, SSN, WS: Data collection

NZ, AA, EA: Conducted data analysis

YAR, MM: Reviewed and edited the manuscript.

YAR: Supervised the manuscript.

## Figures and Tables

**Table 1.** Demographics.

Variable	Total No/ Percentage %
Gender • Male • Female	22 (43.1%) 29 (56.8%)
Location of metastatic disease • Lungs • Hepatic • Nodal • Brain • Bony • Adrenal • Prostate	51 (100%) 37 (72.5%) 41 (80.3%) 41 (80.3%) 16 (31.3%) 1 (1.9%) 1(1.9%)
The stage at the time of diagnosis • Stage I • Stage II • Stage III • Stage IV	0 01 (2%) 0 50 (98%)
Smoking	10 (19.6%)
Packs of cigarette 10 or more 20 or more 30 or more 100 or more Not known Non smokers	02 02 02 01 01 41 (80.4%)
Family history of cancer	06 (11%)
ECOG performance status • 1 • 2 • 3 • 4	26 (50.9%) 14 (27.4%) 09 (17.6%) 02 (3.9%)
Pleural fluid cytology • Negative • Positive • Not done	11 (21.5%) 13 (25.4%) 27 (52.9%)
Histopathology • Adenocarcinoma	51 (100%)
Type of mutation • Exon 19 deletion • Exon 20 Insertion • Exon 21 • Compound • Others • T790M	31 (60.7%) 05 (9.8%) 10 (19.6%) 04 (7%) 01 (1.8%) 02 (3.9%)
History of previous oncological treatment	01 (2%)
Targeted therapy	42
Type of targeted therapy • Erlotinib • Afatinib • Osimertinib • Did not get therapy	18 (35.2%) 01(1.8%) 23 (45%) 09 (17.6%)
Dose reduction • Yes • Not needed	06 (14.2%) 36 (85.7%)
Treatment response • Disease progression • Stable disease • Not needed	37 (72.5%) 12 (23%) 02 (4%)

**Table 2.** Univariate analysis.

Variable name	Significance (Sig.)	Hazards ratio	Lower 95% CI	Upper 95% CI
Performance status (ECOG)	0.471	0.015	0.000	1,327.440
Mutation	0.01	0.178	0.000	2,712.892
Type of mutation	0.725	0.178	0.000	2,712.892
Type of targeted therapy?	0.616	434.450	0.000	8,953,863,135,600.588
Stage at diagnosis?	0.616	0.048	0.000	6,887.566
Location of disease?	0.725	0.178	0.000	2,712.892
Gender	0.616	434.450	0.000	8,953,863,135,600.588
Pleural fluid cytology	0.464	6.458	0.044	947.343
Mutation detected	0.01	0.17	0.000	2,712.892

**Table 3.** Toxicity profile.

Serial No	Adverse events	Any grade (%)	Grade II	Grade III
1.	Skin rash	6 (11.7%)	1	5
2.	Diarrhea	0	0	0
3.	Nausea	0	0	0
4.	Vomiting	0	0	0
5.	Neutropenia	0	0	0
6.	Fever	0	0	0
7.	Abnormal liver function test	0	0	0
